# Leveraging teleradiology with artificial intelligence

**DOI:** 10.2471/BLT.25.020225

**Published:** 2025-02-01

## Abstract

Can teleradiology supported by artificial intelligence (AI) boost overall radiology capacity in resource-constrained settings? Gary Humphreys reports.

The pictures taken by Prashant Warier’s team when they reached Everest base camp in Nepal’s Khumbu region in April of 2022 were not the usual kind. Instead of sweeping images of the still-distant summit or the Khumbu glacier, they got some extreme close-ups of people’s lungs.

“The images were captured on a portable, battery-powered digital radiography unit, weighing less than 30 kilos, then scanned with an AI-supported diagnostic tool,” says Warier, the founder and chief executive officer of a digital health company based in Mumbai, India.

Prior to arriving at Everest base camp, Warier’s team had passed through villages along the trekking route, conducting screenings on local residents and trekkers with their informed consent. “We were able to provide real-time diagnostics for tuberculosis, pulmonary oedema and a range of other issues,” Warier explains.

Teleradiology is one of the oldest and most widely used telemedicine specialties, with initiatives dating back to the late 1990s. But it is getting increased attention with the addition of AI-supported functionality, ranging from the management of archives used to store, retrieve, manage and share medical imaging files to diagnostic analysis of images.

“Over the past five years, several studies have demonstrated AI’s potential in areas such as medical imaging screening and system management,” says Salim Azzabi Zouraq, a digital health expert at the World Health Organization (WHO).

He points out, however, that fully harnessing this potential requires addressing challenges that range from infrastructure and regulation to bias and ethical considerations, and adds, “We are still far from considering it as a replacement for doctors”, underlining concerns around accuracy, bias, accountability and ethics.

But even without replacing actual radiologists, AI-supported radiology can make a huge difference to health systems. At least this is the argument made by Warier and innovators like him, who point out that just by providing a reliable first look, AI-supported systems can address the dearth of actual radiologists.

That there is a dearth is not disputed. Estimates vary as to its size, but according to the International Atomic Energy Agency's IMAGINE database, which compiles data on medical imaging and nuclear medicine resources from over 170 countries, some 309 642 radiologists are active globally, 103 298 in high-income countries (home to 1.2 billion people), 48 444 in lower-middle-income countries (home to 3.3 billion people) and just 910 in low-income countries (home to 702 million people). In other words, low-income countries make do with an estimated 1.3 radiologists per 1 million people, compared to 85.3 per million in high-income countries.

“The tools […] can be used to identify the most serious cases.”

Prashant Warier

Warier wants to change that by filtering out the kinds of cases that take up radiologists’ time, but do not result in referrals. “The kinds of tools we have developed can be used to identify the most serious cases, directing those to human radiologists who then make a determination,” he explains.

Other innovators are using AI-supported screening to connect with human radiologists outside resource-constrained systems. Amr Abodraiaa, the co-founder and CEO of an AI-powered teleradiology platform based in Cairo, Egypt, is one.

According to Abodraiaa, health-care facilities upload medical images along with patient information to the platform. The company’s AI system then screens the images and matches each case with the most suitable radiologist – considering specialization, availability and urgency – from a network of 150 experts located worldwide.

“The radiologist remotely accesses the images and patient data, analyses the findings, and prepares a diagnostic report, which is promptly delivered back to the health-care facility,” Abodraiaa explains.

Both the Cairo- and Mumbai-based companies have obtained stamps of approval from regulatory authorities, including regulatory authorities in Europe and the United States of America, underscoring the quality of the products and processes they are selling; but to what extent can they be rolled out in lower-income countries?

Dr Caroline Perrin Franck believes that the current practical impact of AI-supported teleradiology may not yet match its supporters’ hopes. Executive director of the Geneva Digital Health Hub at Geneva University, Perrin works with the *Réseau en Afrique Francophone pour la Télémédecine* (RAFT), a collaborative telemedicine initiative by Geneva University Hospitals.

RAFT works with hospitals, universities, governments and international organizations, using telemedicine and digital tools, including teleradiology, to improve health-care access, education and collaboration. As a result, Perrin sees implementation challenges up close, starting with limited technological infrastructure. 

The challenges begin with capturing high quality images, given the lack of technicians required to operate the relevant machines, and the lack of maintenance staff required to keep them running. And once captured, those images need to be stored. “Cloud-based platforms are emerging as an enabler of teleradiology in low- and middle-income countries, allowing facilities to share radiological images without costly on-site infrastructure, but stable internet connectivity remains a problem, especially in remote regions with unreliable networks, alongside security and interoperability issues,” she says.

For Azzabi Zouraq, the quality of AI is a key concern, with one of the biggest challenges being the lack of relevant training data for its algorithms. “AI algorithms require large amounts of contextually relevant data to function effectively as screening and diagnostic tools,” he explains.

To effectively identify variations and detect anomalies, algorithms perform best when trained on data that accurately reflects the population they are designed to serve. According to Azzabi Zouraq, countries that have invested in big data and the supporting infrastructure will be better equipped to advance AI-driven teleradiology in the future.

Bias is another concern. “If the training data predominantly features images from a specific population, the AI may not generalize well to diverse patient groups,” Azzabi Zouraq says. “For example, an AI trained primarily on data from one region or demographic might face challenges when applied to other populations due to differences in patient characteristics.”

For his part, Warier believes that it’s possible to overstate the significance of disparities between populations and institutional sources. “We have trained our AI on data sets from multiple sources and have not encountered significant problems,” he asserts.

Warier is also quick to point out that AI’s value-added is not limited to boosting screening capacity. His company also uses AI to enhance data management – for example by automating data collection, organizing imaging results and providing real-time reporting.

“The challenges faced in […] resource-constrained settings need to be understood.”

Salim Azzabi Zouraq

Additionally, AI is used to facilitate referrals by flagging cases for specialist care and enabling communication between providers and referral centres. In community health programmes, mobile units identify at-risk patients for timely diagnosis and follow-up.

While such support can help – what difference can it make in health systems lacking the services required to follow through with referrals? For Perrin, this is one of the biggest challenges faced. “The inadequacy of health systems significantly prevents AI-supported teleradiology realizing its full potential,” she says. “Without adequate personnel to operate the tools and interpret results, the technology cannot be effectively utilized; and the same is true of the specialists needed to receive any referrals flowing through the system.”

Perrin emphasizes that while AI-supported teleradiology holds immense promise, its success depends on addressing these systemic limitations. She argues that efforts must focus on strengthening infrastructure, building workforce capacity, and ensuring the necessary policies are in place to create an environment where AI tools can be effectively deployed.

Azzabi Zouraq shares Perrin’s assessment, noting that WHO’s work in this area includes the publications designed to promote the adoption of digital health tools and strengthen health-care systems to achieve universal health coverage. These include the WHO *Global strategy on digital health 2020–2025*, which provides a strategic vision for leveraging digital innovations, and a toolset designed to translate WHO recommendations into machine-readable, adaptive digital interventions.

“While technological advancements offer promising solutions, the challenges faced in deploying such systems in resource-constrained settings need to be understood,” Azzabi Zouraq says.

Resource-constrained settings like the sides of mountains. Warier chose Everest base camp, precisely because it was cut off from health services. “The point of the exercise was to capture an image, screen it and deliver a diagnostic result in real time,” he says. Fortunately, none of the volunteers screened required any follow-up. Had that not been the case, they would have needed to access Nepal’s health system, with all the challenges that would have implied.

“For teleradiology to realize its potential, it must be integrated into broader health-care strategies that address infrastructure and personnel gaps, ensure equitable access, and build local capacity,” says Azzabi Zouraq.

There is still a mountain to climb.

**Figure:**
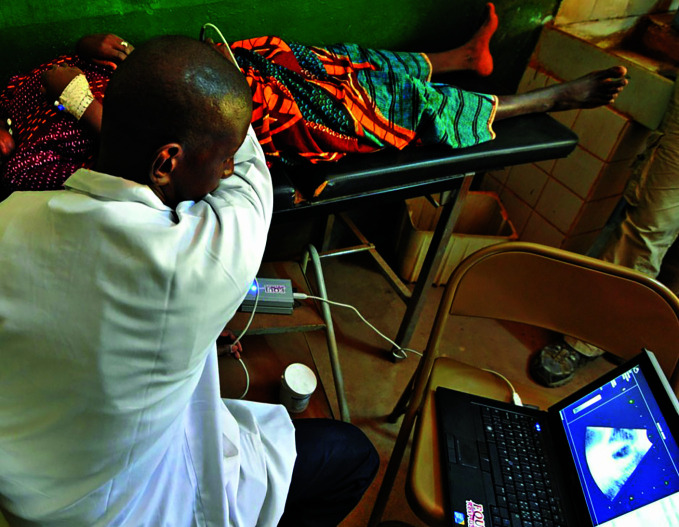
Conducting a teleradiology examination in Mali

Courtesy of Antoine Geissbuhler

